# Association between childhood trauma and multimodal early-onset hallucinations

**DOI:** 10.1192/bjp.2019.266

**Published:** 2020-03

**Authors:** François Medjkane, Charles-Edouard Notredame, Lucie Sharkey, Fabien D'Hondt, Guillaume Vaiva, Renaud Jardri

**Affiliations:** 1 Child Psychiatrist, Clinical Director, PSY team, Centre Lille Neuroscience & Cognition, INSERM U1172, Univ Lille; and Department of Child and Adolescent Psychiatry, Fontan Hospital, CHU Lille, France; 2 Child Psychiatrist, PSY team, Centre Lille Neuroscience & Cognition, INSERM U1172, Univ Lille; and Department of Child and Adolescent Psychiatry, Fontan Hospital, CHU Lille, France; 3 Psychologist, CHESS Hallucination Clinic & Reference Centre for Rare Diseases with Psychiatric Manifestations, Department of Child and Adolescent Psychiatry, Fontan Hospital, CHU Lille, France; 4 Associate Professor in Neuroscience, PSY team, Centre Lille Neuroscience & Cognition, INSERM U1172, Univ Lille; and National Centre for Resource and Resilience (CN2R), Lille and Paris, France; 5 Professor of Adult Psychiatry, PSY team, Centre Lille Neuroscience & Cognition, INSERM U1172, Univ Lille; and National Centre for Resource and Resilience (CN2R), Lille and Paris, France; 6 Professor of Child and Adolescent Psychiatry, Clinical & Research Director, PSY team, Centre Lille Neuroscience & Cognition, INSERM U1172, Univ Lille; and CHESS Hallucination Clinic & Reference Centre for Rare Diseases with Psychiatric Manifestations, Department of Child and Adolescent Psychiatry, Fontan Hospital, CHU Lille, France

**Keywords:** Hallucinations, voice hearing, childhood trauma, multisensory, phenomenology

## Abstract

Previous reports suggest that adverse events during childhood could be related to an array of psychiatric problems. Here, we question the relationship between childhood traumatic experiences and the sensory complexity of hallucinations in a cohort of 75 children and adolescents. We evidence a positive link between the number of sensory modalities involved in hallucinations and history of childhood trauma, even after controlling for the co-occurrence of suicidal ideation or the number of ICD-10 diagnoses. These findings support initiatives in which a routine exploration of traumatic events in childhood is performed when multimodal hallucinations are present.

Recurrent evidence suggests that adverse childhood experiences are associated with a wide range of negative outcomes, including suicidal behaviour in adolescents,^1^ and are related to a greater risk for psychiatric disorders. In one meta-analysis, early trauma was shown to substantially increase the risk of psychotic experiences, with an odds ratio (OR) of 2.8.^2^ The significance of the type of trauma is sometimes mentioned, with potentially higher impact for rape and physical abuse.^3^ However, this specific link was not confirmed in subsequent meta-analysis,^2^ which was more supportive of a broad measurement of trauma history. Regarding reported psychotic experiences, paranoia and auditory verbal hallucinations were the most prominent. A non-linear dose–effect relationship between auditory hallucinations and the number of traumatic events was notably evidenced,^3^ pointing to the importance of a thorough assessment and dedicated care for such experiences. Interestingly, it has been proposed that hallucinations could constitute a clinically accessible probe for past traumatic events.

The underlying mechanisms linking trauma to hallucinations are still unclear. This question probably suffers from a high proportion of retrospective designs susceptible to recall bias and/or confounding, but also from the lack of dedicated tools to investigate trauma history and precisely explore hallucinations in young people. The debate would gain by focusing on particular features of early-onset hallucinations, beyond a strict symptom-severity approach. In this vein, some authors recently pointed out the value of more subtle phenomenological properties of hallucinations to decipher their mechanisms.^4^ Despite the high occurrence of voices during development, even before 6 years of age,^5^ the multisensory nature of experiences and their vividness were mentioned as particularly interesting properties in paediatric clinical populations.^6^ The number of sensory modalities in hallucinations was even proposed as a proxy of the developmental vulnerability to hallucinations.^7^ However, until now, no scientific report specifically explored the relationship between the multimodality of hallucinatory experiences and childhood trauma. For the first time, we question whether the number of sensory modalities involved in early-onset hallucinations as identified in clinical settings carries information regarding the probability a given child ever experienced previous traumatic events.

## Method

We assessed data from 75 children and adolescents who attended the CHESS clinic, an out-patient clinic for distressing early-onset hallucinatory experiences in Lille, France, between January 2017 and June 2019. Among the collected clinical variables, we systematically screened for past traumatic experiences, defined as a history of (a) physical, verbal or emotional abuse (including bullying), (b) neglect or (c) household dysfunction (such as substance misuse or domestic violence). The presence of suicidal ideation and total number of psychiatric and medical diagnoses per participant were based on standardised ICD-10 coding in individual medical records. We finally determined the number of modalities involved in hallucinatory experiences using the Multisensory Hallucinations Scale for Children (MHASC).^8^ The collected sociodemographic and clinical data are reported Table 1. These data were anonymously used in accordance with European General Data Protection Regulation legislation and after collecting written consent from the participants and their parents (CNIL-DEC2015-152 authorisation).

**Table 1:** Sociodemographic and clinical characteristics of the CHESS cohort (*n* = 75)

	Values
Age, years: mean (s.d.) [range]	12.1 (3.4) [2–18]
Gender, female: *n* (%)	25 (33.3%)
Educational level,^a^ mean (s.d.) [range]	1.9 (0.7) [0–3]
Presence of a sensory deficit, *n* (%)	5 (6.7%)
Intellectual disability,^b^ *n* (%)	13 (17.3%)
History of childhood trauma, *n* (%)	11 (14.7%)
Number of ICD-10 diagnoses, mean (s.d.) [range]	1.6 (0.9) [0–4]
Total CBCL score,^c^ mean (s.d.) [range]	44.3 (33) [18–122]
Number of hallucinatory modalities, mean (s.d.) [range]	2 (1.1) [0–5]
Presence of suicidal ideas, *n* (%)	16 (21.3%)
Prior treatments,^d^ *n* (%)	32 (42.7%)

a. Based on the 2011 International Standard Classification of Education (ISCED 2011).

b. Defined as a Wechsler Intelligence Scale for Children, 5th edition (WISC-V) IQ score <70.

c. Child Behavior Checklist for Ages 6–18.

d. Active psychiatric monitoring (including prior psychotropic medication in 28.1% of cases) or psychotherapy sessions.

Analyses were conducted using the JAMOVI v1.1.4 software package for Windows. To determine to what extent sensory modalities could predict the probability of a previous early trauma, we conducted a hierarchical binomial logistic regression with history of childhood trauma as the binary dependent variable in three different models. In the first model, we introduced control variables (i.e., age, educational level and number of ICD-10 diagnoses). In the second, the presence of suicidal ideation, known to be associated with both hallucinations and early trauma, was introduced as an independent variable. Finally, the third model assessed whether adding the number of hallucinatory modalities as a new variable was able to explain additional variance.

## Results

The mean age of the 75 participants was 12.1 years (s.d. = 3.4) and 33% were female. In total, 21.3% expressed suicidal ideation and 14.7% reported childhood trauma exposure; 62.7% reported multisensory hallucinations, with a majority of audio-visual experiences (36.0%), followed by unisensory experiences (34.7%, mainly auditory), then hallucinations involving three (17.3%), four (5.3%) or five (4.0%) sensory modalities. Twenty-two different ICD-10 diagnoses were made (ranging from 0 to 4 per participant). The five most common were: major depressive disorder (16.0%), borderline personality disorder (10.7%), childhood-onset schizophrenia (9.3%), post-traumatic stress disorder (PTSD) (8.0%) and attention-deficit hyperactivity disorder (5.3%). No medical diagnosis was established in 10.7% of the sample. See Table 1 for a full description.

**Fig. 1:**
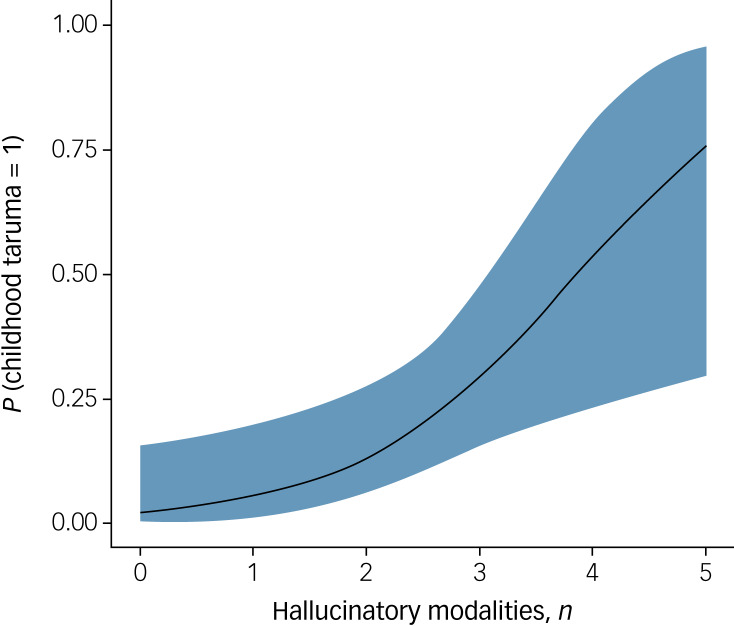
Estimated marginal means with 95% confidence interval showing the positive relationship between the number of sensory modalities in hallucinatory experiences and the probability of a childhood trauma (OR = 2.24 (95% CI 1.16–4.33), *P* = 0.017, *n* = 75).

After checking for the assumption of collinearity, we found that the third logistic regression model outperformed the two others. This was true when using model fit measures (such as Bayesian information criteria scores, which penalise model complexity) and using more direct model comparisons (χ^2^ = 6.02, *P* = 0.014). The overall model evaluation was *R*^2^ = 0.350, χ^2^ = 16.53, *P* = 0.005. The number of hallucinatory modalities appeared to be the only significant predictor of childhood trauma (estimate (i.e. the log odds of ‘history of childhood trauma’ = 1 *v.* 0) 0.81, s.e. = 0.34, *z* = 2.39, *P* = 0.017, OR = 2.24 (95% CI 1.16–4.33); see Fig. 1). Other variables’ estimates were not significant.

## Discussion

Our findings suggest a strong link between the sensory complexity of early-onset hallucinations and history of childhood trauma, even after controlling for the co-occurrence of suicidal ideation or the number of ICD-10 diagnoses. Associations between hallucinations' severity and trauma have previously been established,^2,3^ but this report is the first to emphasise a specific relationship between trauma and the number of sensory modalities. At this stage, the exact nature of the mechanisms involved remains unclear. Should the hypothesis that multimodal hallucinations reflect a developmental vulnerability to hallucinations^7^ be proven right, and given the possible commonalities in the neurobiological changes induced by trauma and those associated with hallucinations,^9^ future studies on the CHESS cohort will have to address this issue and specify the effect on this ‘trauma–multisensory hallucinations’ association of (a) complex trauma accumulation during childhood and (b) exposure during specific developmental periods.

This study also underscores the need to consider trauma-related symptoms in youth beyond a strict PTSD diagnosis. Although preliminary, our results support initiatives in which a routine exploration of traumatic events in childhood is performed when multimodal hallucinations are present, in complement to other pertinent features such as help-seeking.^10^ Because young people rarely volunteer information about these intimate experiences, dedicated tools able to non-intrusively explore the phenomenology of early-onset hallucinations could be particularly helpful^8^ and allow clinicians to better define adjusted and personalised healthcare.

In this vein, trauma-informed practice should maximise a sense of safety in young people with multimodal hallucinations, while minimising exposure to situations able to evoke traumatic memories and trigger hallucinatory experiences. In a global context of fast-growing causes of stress, from extreme weather events to forced migration and terrorist threats, this strategy appears perfectly in line with recent French public policy aimed at (a) providing research support and (b) promoting evidenced-based care for trauma victims, thanks to the structuring and collaboration of mental health services at the regional and national level.

## Data Availability

The anonymised data-set is available from the corresponding author on reasonable request.
